# Leaf-associated macroinvertebrate assemblage and leaf litter breakdown in headwater streams depend on local riparian vegetation

**DOI:** 10.1007/s10750-022-05049-7

**Published:** 2022-10-18

**Authors:** Rebecca Oester, Paula C. dos Reis Oliveira, Marcelo S. Moretti, Florian Altermatt, Andreas Bruder

**Affiliations:** 1 Institute of Microbiology, University of Applied Sciences and Arts of Southern Switzerland, via Flora Ruchat Roncati 15, 6850 Mendrisio, Switzerland; 2 Department of Evolutionary Biology and Environmental Studies, University of Zurich, Winterthurerstr. 190, 8057 Zurich, Switzerland; 3 Department of Aquatic Ecology, Eawag: Swiss Federal Institute of Aquatic Science and Technology, Überlandstrasse 133, 8600 Dübendorf, Switzerland; 4 Laboratory of Aquatic Insect Ecology, Universidade Vila Velha, Av. Comissário José Dantas de Melo 21, Vila Velha, ES 29102-920 Brazil

**Keywords:** Leaf decomposition, Fragmentation rate, Forested stream, Terrestrial-aquatic linkages, Ecosystem functioning, Detrital food web

## Abstract

Headwater streams harbor diverse macroinvertebrate communities and are hotspots for leaf litter breakdown. The process of leaf litter breakdown mediated by macroinvertebrates forms an important link between terrestrial and aquatic ecosystems. Yet, how the vegetation type in the local riparian zone influences leaf-associated macroinvertebrate assemblages and leaf litter breakdown rates is still not resolved. We investigated how leaf-associated macroinvertebrate assemblages and leaf litter fragmentation rates differ between forested and non-forested sites using experimental leaf litter bags in sixteen sites paired across eight headwater streams in Switzerland. Our results show that sensitive taxa of the invertebrate orders Ephemeroptera, Plecoptera and Trichoptera (EPT) and the functional group of shredders were strongly associated with forested sites with overall higher values of abundance, diversity, and biomass of EPTs in forested compared to non-forested sites. However, the importance of riparian vegetation differed between study regions, especially for shredders. Fragmentation rates, which are primarily the result of macroinvertebrate shredding, were on average three times higher in forested compared to non-forested sites. Our results demonstrate that not only the composition of the aquatic fauna but also the functioning of an essential ecosystem process depend on the vegetation type in the local riparian zone.

**Supplementary Information:**

The online version contains supplementary material available at 10.1007/s10750-022-05049-7.

## Introduction

Headwater streams are tightly connected to their terrestrial surrounding (Richardson & Danehy, 2007; Little & Altermatt, 2018; Riis et al., 2020). Under natural conditions, many headwater streams flow through forests and other areas with woody riparian vegetation. Forests in the riparian zone not only provide shade to streams but also inputs of carbon and nutrients in the form of dead plant material (Fisher & Likens, 1973; Wallace et al., 1997; Gounand et al., 2018). Most headwater streams can therefore be considered as primarily net heterotrophic (Marcarelli et al., 2011). As stream communities depend on the input of terrestrial organic matter, they are sensitive to changes in the composition and structure of the riparian vegetation (England & Rosemond, 2004; Burdon et al., 2020b).

The vegetation type in the riparian zone can affect the composition of aquatic communities (Allan, 2004; Sweeney & Newbold, 2014; Little & Altermatt, 2018; dos Reis Oliveira et al., 2020). This is particularly true for organism groups that inhabit both aquatic and terrestrial ecosystems, such as many macroinvertebrates (Cummins et al., 1989; Clarke et al., 2008). For example, as aquatic larvae, many insect species depend on leaf litter as food resource (i.e., shredders), substrate, or shelter (Graça, 2001; Moretti et al., 2009; Mendes et al., 2017). Such leaf-associated macroinvertebrates thus depend directly on the quantity (Hall et al., 2000) and quality (Marcarelli et al., 2011; Handa et al., 2014) of terrestrial plant material provided by the riparian vegetation (Iñiguez-Armijos et al., 2018; Estévez et al., 2020). Especially sensitive taxa as those of the orders Ephemeroptera, Plecoptera, and Trichoptera (EPT) also indirectly respond to the environmental conditions influenced by the riparian vegetation including water quality (Goss et al., 2014) or shading (Li & Dudgeon, 2008; Lagrue et al., 2011). As winged adults, many aquatic insect taxa seek refuge in the riparian vegetation (Reinhart & VandeVoort, 2006; Yoshimura, 2012) and use it during mating, dispersal and feeding (Jackson & Resh, 1989; Sweeney, 1993). Land-use practices affecting the structure and composition of the riparian vegetation can therefore alter these cross-ecosystem linkages and the two mutually non-exclusive macroinvertebrate groups of EPTs and shredders (Gücker et al., 2009). To assess the consequences of land-use and management practices around streams, it is important to better understand how aquatic communities are connected to local riparian vegetation in these “small but mighty” ecosystems (Finn et al., 2011).

Macroinvertebrates, and particularly shredders, contribute substantially to the breakdown of allochthonous plant material and the propagation of this resource in stream food webs (Wallace et al., 1982; Hieber & Gessner, 2002). Shredders feed on and fragment coarse particulate organic matter (CPOM) and thereby transform a considerable part of it into fine particulate organic matter (FPOM). FPOM, in turn, is an important food source for other macroinvertebrates and microorganisms (Cummins et al., 1989). The rate at which macroinvertebrate communities fragment CPOM can be expressed as fragmentation rate comprising both physical abrasion and macroinvertebrate feeding (Lecerf, 2017). Only few studies have calculated fragmentation rate as a distinct component of the overall process of leaf litter breakdown (Lecerf, 2017; Yeung et al., 2018; Omoniyi et al., 2021). However, fragmentation rates likely vary with shredder abundance, diversity, and biomass, as overall leaf litter breakdown rates have been associated with these community metrics (Wallace & Webster, 1996; Jonsson et al., 2001; Hieber & Gessner, 2002; Iñiguez‐Armijos et al., 2016).

In this study, we investigated the effects of the presence and absence of forests in the local riparian zone on both leaf-associated macroinvertebrate assemblages and leaf fragmentation rates. Firstly, we studied how leaf-associated macroinvertebrate assemblages in experimental leaf litter bags differed between forested and non-forested sites, with focus on the taxonomic and functional sub-groups of EPT and shredders, respectively. Secondly, we studied the effects of riparian vegetation on abundance, diversity and biomass of EPTs and shredders and whether observed patterns were consistent across two distinct biogeographic regions north and south of the Swiss Alps, differing in geology and chemical composition of the water. Thirdly, we studied how leaf litter fragmentation rates were associated with forested and non-forested sites and whether patterns in fragmentation rates differed among leaves from different tree species. To do so, we carried out a field experiment using leaf litter bags in 16 different sites paired in eight headwater streams in northern and southern Switzerland. In each stream, we studied pairs of sites surrounded by vegetation types of either natural mixtures of deciduous trees (forested) or mainly grasslands (non-forested). In forested sites, we expected higher abundances, diversity, and biomass of EPT and shredders in the leaf litter bags as well as higher leaf litter fragmentation rates compared to non-forested sites independent of biogeographic regions. Further, we expected similar effect sizes of riparian vegetation and of leaf treatment. Testing these hypotheses allowed us to increase our understanding on the consequences of common land-use and management practices in the local riparian zone on headwater streams, their communities, and a key ecosystem process.

## Materials and methods

### Experimental design

**Fig. 1:**
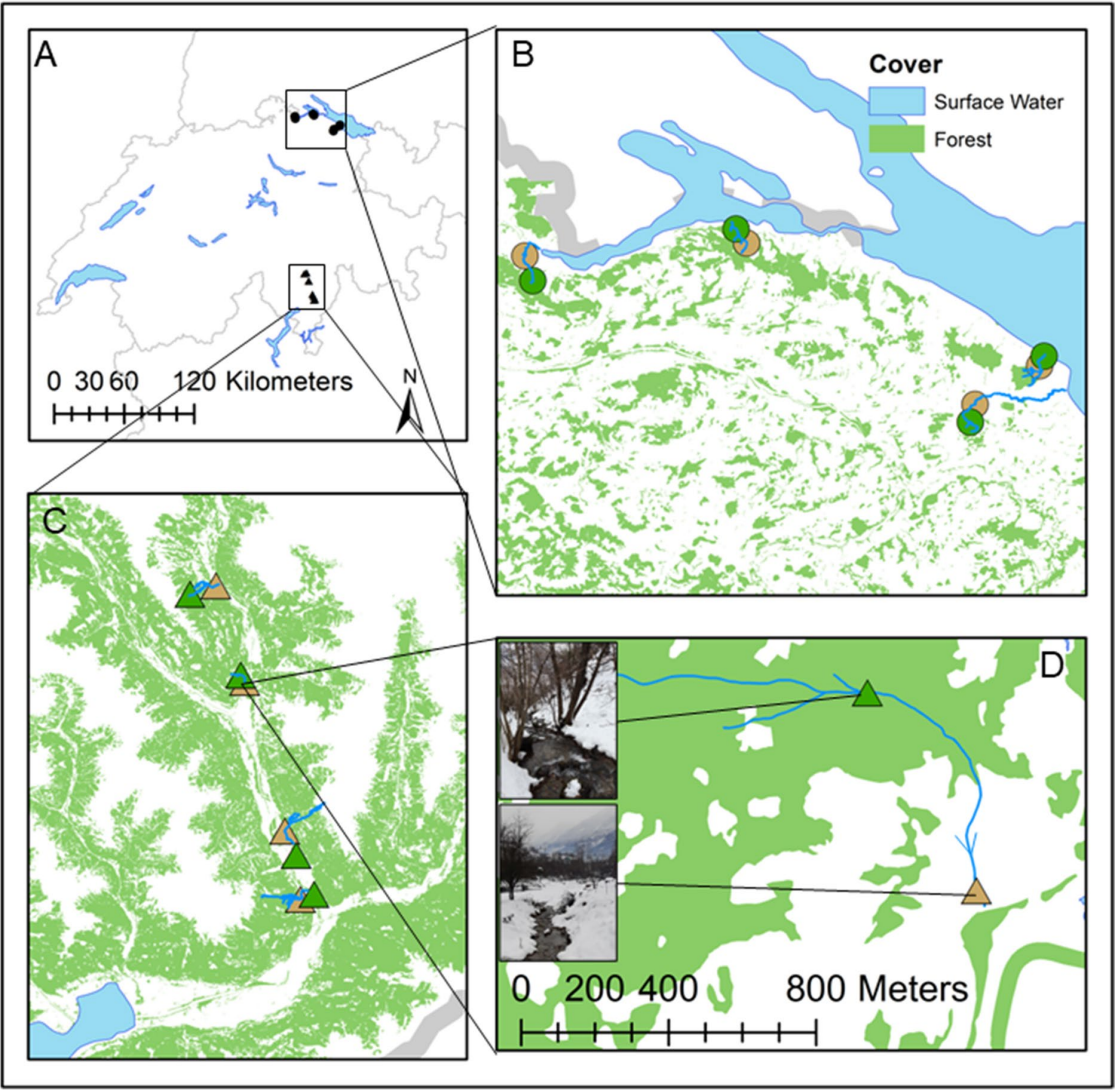
Locations of the eight headwater streams. **A** Map with the two study regions in Switzerland, **B** headwater streams in the northern region (circles), **C** headwater streams in the southern region (triangles), **D** a headwater stream showing a paired forested (green triangle) and a non-forested (brown triangle) site. Data provided by Swisstopo, (2007, 2010) and map produced with ArcGis Map (version 10.7.1, ESRI)

We selected eight streams in Switzerland, each having a distinct section with the riparian vegetation consisting of densely standing trees (forested) and another section surrounded by grassland or extensively used pasture with no or only isolated trees or bushes (non-forested). The forested site was located in the upstream section in half of the streams and in the downstream section in the other half, which resulted in a balanced and paired comparison between forested and non-forested sites within the streams. Four streams were situated in the north of Switzerland and four in the south (Fig. 1). The river distance between each two sites within the stream was on average around 500 m (± 250 m), with slightly longer river distances in the northern region (Table S1). The sites of the eight streams had, other than the forest-non-forest transition, only minimal anthropogenic disturbances or modifications and an upstream catchment dominated by forest cover (Table 1; Table S1).

**Table 1:** Site characteristics and water parameters with values representing means ± 1SD grouped by region and riparian vegetation

Region	North		South	
Riparian vegetation	Forested	Non-forested	Forested	Non-forested
Elevation (m a.s.l.)	487.62 ± 74.27	467.35 ± 40.57	417.22 ± 241.54	390.42 ± 211.12
Catchment area (km^2^)	1.98 ± 1.02	2.34 ± 1.96	3.15 ± 2.18	3.64 ± 1.84
Width (cm)	183.34 ± 62.55	93.58 ± 26.12	304.82 ± 173.14	274.44 ± 131.16
Depth (cm)	10.74 ± 2.6	15.09 ± 7.15	13.70 ± 3.78	17.48 ± 1.8
Velocity (m s^−1^)	0.18 ± 0.05	0.20 ± 0.05	0.21 ± 0.05	0.27 ± 0.15
Riparian cover (%)	31.10 ± 3.52	7.86 ± 6.47	29.10 ± 5.39	5.54 ± 5.87
Temperature (°C)	5.13 ± 0.73	5.58 ± 0.90	3.30 ± 0.59	3.26 ± 0.40
Nitrate (mg l^−1^)	4.24 ± 1.62	3.76 ± 2.24	0.66 ± 0.13	0.70 ± 0.14
Phosphate (μg l^−1^)	3.98 ± 2.25	3.84 ± 1.19	3.67 ± 1.95	11.89 ± 11.35
DOC (mg l^−1^)	3.75 ± 2.55	3.82 ± 2.69	1.55 ± 0.53	1.66 ± 0.66
pH	7.11 ± 0.10	7.19 ± 0.14	8.06 ± 0.10	7.94 ± 0.30
Conductivity (μS cm^−1^)	319.25 ± 55.2	322.75 ± 68.51	60.75 ± 21.53	70.75 ± 32.35
Oxygen saturation (%)	97.74 ± 0.77	97.69 ± 2.47	100.15 ± 0.40	100.89 ± 1.48

To determine leaf-associated macroinvertebrate assemblages and the rate at which macroinvertebrates fragment leaf litter, we used experimental leaf litter bags with two different mesh sizes that either allowed (coarse-mesh bags) or prevented (fine-mesh bags) macroinvertebrate access to the leaves. In each of the 16 sites, we deployed eight coarse-mesh (10 mm mesh size) and eight fine-mesh (0.25 mm mesh size) bags. We used 5 g (SD = 0.03 g) of dried leaf litter (at 40°C for 48 h) from either black alder (*Alnus glutinosa* (L.) Gaertn*.*) or European ash (*Fraxinus excelsior* L.) representing palatable leaf litter from common riparian tree species (Bruder et al., 2014). Leaves from alder and ash differ in their C and N content with alder being a N-fixing species and therefore having a lower C:N ratio compared to ash (Hladyz et al., 2009). Each combination of mesh size and leaf treatment was replicated four times within each study site, which resulted in 16 leaf litter bags per site and a total of 256 leaf litter bags. At each site, we placed the four replicate treatments within a 20 m section. We removed the leaf litter bags when approximately 50% of the initial leaf litter mass of the faster decomposing species (ash) was lost. Consequently, the duration of the field experiment varied among region resulting in 3 weeks in the northern region and 5 weeks in the southern region between December 2020 and January 2021.

### Stream characterization

We measured physical and chemical parameters of the stream, stream hydromorphology and relevant aspects of the riparian vegetation (Table 1; Fig. S1; Table S1) at each site. Water temperature was measured every hour using data loggers (HOBO Temperature DataLogger; UA-002-64; Onset Computer Corporation). At the beginning and end of the experiment we measured pH, dissolved oxygen saturation in the field (HQ40d multi sonde, Hach), and took water samples to measure nitrate, phosphate and dissolved organic carbon (DOC) concentrations, and electrical conductivity. To evaluate stream hydromorphology, we measured flow velocity (MiniAir20, Schildknecht) in front of each coarse-mesh bag, and width, depth, and flow velocity at ten locations within each site. We characterized substrate cover by measuring the proportion of different substrate categories such as sand, gravel, small rocks, and big rocks in a 0.25 m^2^ area repeated ten times per site. To quantify riparian cover, we took fisheye images, converted them to black and white, and calculated the percentage of black pixels on the image in imageJ (National Institute of Health, Bethesda, Maryland, USA; version 1.52c) as proxy for riparian cover. We extracted the watercourses from the Swiss national 1:25,000 scale water network (Swisstopo, 2007) and used it to determine the distance along the watercourse between upstream and downstream sites within a stream. We also extracted the catchment area for each site and the land cover (BAFU, 2008) using ArcGIS version 10.7.1 (ESRI, Redlands, California, USA).

### Leaf-associated macroinvertebrate assemblage

To retain the macroinvertebrates in the leaf litter bags at the end of the experiment, we carefully detached the bags and placed them individually into plastic bags under water. In the laboratory, we washed the leaf litter over a 250 μm sieve and preserved the collected macroinvertebrates in ethanol (98%). We later sorted, counted, and identified the macroinvertebrates under a dissecting microscope to the lowest taxonomic level possible using determination keys (e.g., Sundermann et al., 2007; Tachet et al., 2010). To determine functional feeding groups, we assigned feeding preferences to all taxa based on the freshwaterecology.info database (Schmidt-Kloiber & Hering, 2015; version 8.0; accessed on 01 October 2021). We averaged the trait values across the respective taxonomic level over a fuzzy code ranging from 0 to 10 in the categories: grazers/scrapers, miners, xylophagous taxa, shredders, gatherers/collectors, active filter feeders, passive filter feeders, predators, parasites and other feeding types (Moog, 1995). We combined the abundance data and the feeding preferences to community-weighted mean trait (CWMT) values by multiplying the abundance with the values in the ten feeding type categories (ACWMT). The taxa that exhibited their feeding preference as shredding (their maximum value or 5 points) are subsequently referred to as shredders for analyses of community metrics to focus on taxa that rely mostly on CPOM as their primary food source. Additionally, we measured the body size of ten individuals of each taxon per leaf litter bag, averaged these values and calculated an estimated dry weight for all individuals of all taxa in each leaf litter bag using established length–weight regressions (e.g., Benke et al., 1999). We used their dry weight to multiply with the aforementioned feeding preference to create CWMT values based on biomass for each taxon (BCWMT).

### Leaf litter breakdown rates

We calculated leaf litter breakdown rates from fine and coarse-mesh bags based on mass loss during the experiment. Immediately after washing, we dried the leaves of each bag (40°C for 48 h) and calculated leaf litter breakdown rates based on the following exponential decay model:

1 $${m}_{t}={m}_{0}{\text{e}}^{-kt}$$

where *m*_*t*_ is leaf litter dry weight after *t* degree days, m_0_ the initial dry weight and *k* the breakdown rate. We calculated *k*_c_ and *k*_f_ for coarse and fine-mesh bags, respectively. As *k*_c_ contains both microbial and macroinvertebrate-mediated leaf litter breakdown, we additionally calculated the fragmentation rate *λ*_F_ according to Lecerf (2017):

2 $$\lambda_{{\text{F}}} = k_{{\text{c}}} - \frac{{k_{{\text{f}}} - k_{{\text{c}}} }}{{\ln \left( {k_{{\text{f}}} } \right) - \ln \left( {k_{{\text{c}}} } \right)}}$$

This fragmentation rate can be interpreted as the mass loss caused by macroinvertebrate feeding and physical abrasion (Lecerf, 2017; Yeung et al., 2018).

### Statistical analysis

To analyze differences in leaf-associated macroinvertebrate assemblage between forested and non-forested sites and between regions, we used non-metric multidimensional scaling (NMDS). We calculated Bray–Curtis dissimilarities on Hellinger transformed community data of each site (all leaf litter bags per site pooled). We used abundance, ACWMT and BCWMT values to produce these ordinations. We tested for homogeneity of dispersion (PERMDISP) between groups (i.e., riparian vegetation and region) using the betadisper function (Oksanen et al., 2019). As the groups were homogeneously dispersed, we tested for differences in variance with a PERMANOVA (permutational multivariate analysis of variance using distance matrices) using the adonis function of the vegan package (Oksanen et al., 2019). We analyzed the community data in relation to the riparian vegetation and region including their interaction and stream identity as strata.

To further analyze EPT and shredders communities, we calculated their abundance, diversity, and biomass. To calculate the Shannon diversity, we used the diversity function of the vegan package (Oksanen et al., 2019). We quantified correlations between Shannon diversity, abundance, and biomass of EPT and shredders using Kendall’s rank correlation tests. To assess the effects of vegetation type in the riparian zone and region on abundance, Shannon diversity and biomass of EPT taxa and shredders, we used linear-mixed-effect models fit by REML (restricted maximum likelihood) using the nlme package (Pinheiro et al., 2020; version 3.1–149) with the following model structure:

3 $$C\sim V*R+1|{\text{Stream}}$$

where *C* represents the respective community metric of either EPT or shredders (i.e., abundance, Shannon diversity, or biomass), *V* the riparian vegetation (two levels: forested and non-forested) and *R* the region (two levels: north and south). We included stream as a random factor to account for differences of background environmental conditions between sampling locations. Because position of the study site within the streams (two levels: upstream and downstream) did neither substantially increase the fit of the models nor affect the patterns of the test results, we excluded this variable from further analyses.

To test the relationships between fragmentation rates (*λ*_F_) and abundance, Shannon diversity, and biomass of shredders we used three models each with the following structure:

4 $${\lambda }_{\text{F}}\sim C+V+R+L+1|{\text{Stream}}$$

In these models, we used either abundance, Shannon diversity, or biomass of shredders as community metric (*C*), the riparian vegetation (*V*), the region (*R*), and the leaf treatment (*L*) with two levels (alder and ash) as fixed effects and stream as random effect. To evaluate model fit we visually inspected residual plots, and transformed *λ*_F_, abundance, and biomass values with log_10_ to meet model assumptions of homoscedasticity and normality. We calculated marginal and conditional R^2^ for the models using the MuMIn package (Bartoń, 2020).

To further analyze and visualize the overall effects of riparian vegetation on the fragmentation rate (*λ*_F_) we calculated the mean log response ratio [LRR = log(*λ*_F non-forested_/*λ*_F forested_)] between the paired sites of forested and non-forested sections for each stream and leaf treatment. We tested the relationship between riparian vegetation and LRR of fragmentation rate using an unweighted random effect model using the metafor package (Viechtbauer, 2021). All analyses were performed in the statistical software R, version 4.0.3 (R Development Core Team, 2020).

## Results

### Leaf-associated macroinvertebrate assemblage

A total of 22,070 macroinvertebrate individuals were present in the leaf litter bags by the end of the experiment and we distinguished 89 taxa, mostly to genus or species level. We identified twelve taxa that belonged to shredders: *Capnia* sp. (Capniidae), *Nemoura* sp. (Nemouridae), *Protonemura* sp. (Nemouridae), *Athripsodes* sp. (Leptoceridae), Chaetopterygini/Stenophylacini Group Auricollis (Limnephilidae), Chaetopterygini/Stenophylacini Group Permistus, Limnephilini, *Micrasema* sp. (Brachycentridae), *Sericostoma* sp. (Sericostomatidae), *Gammarus* sp*.* (Gammaridae), Hexatomiini (Limoniidae), Limoniini. The overall most abundant shredder taxa belonged to the family of Nemouridae (Plecoptera) ranging from 3 to 900 individuals per site representing on average 78.54% of all shredders per site.

**Fig. 2:**
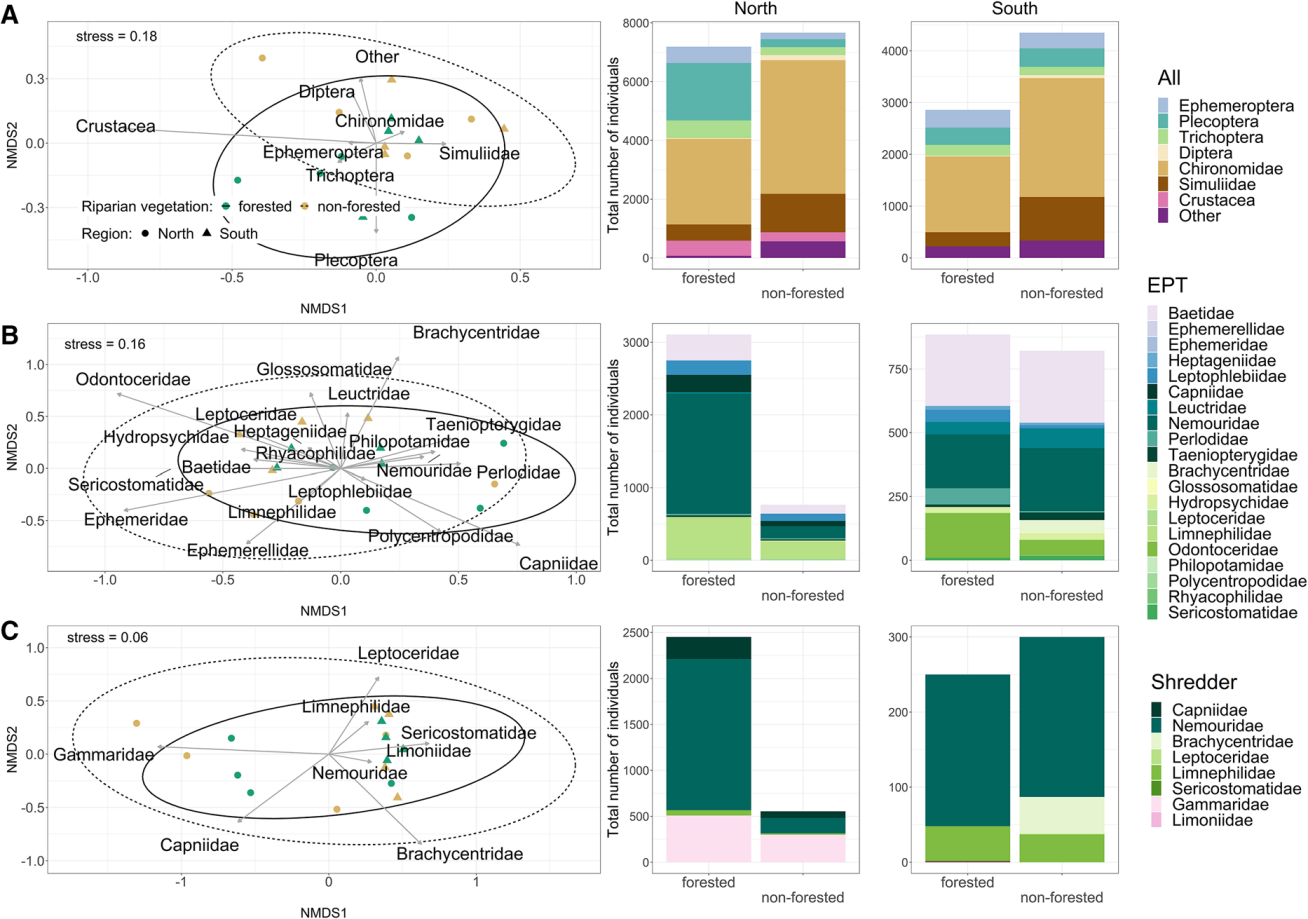
NMDS ordination (*n* = 16) and distribution of taxonomic composition for **A** all macroinvertebrate assemblage, **B** the subset of EPT taxa, and **C** the subset of shredders. For the sake of visibility, only order or family names are shown. In the NMDS plots, green symbols indicate forested sites, brown symbols non-forested sites, circles represent northern sites and triangles southern sites. Solid ellipses encircle communities from forested streams, and dashed ellipses enclose those from non-forested streams (95% confidence interval). The bar plots represent the cumulative number of individuals for each group. “Other” include taxa from Bivalvia, Coleoptera, Gastropoda, Hirudinea, Hydracarina, Nematoda, Odonata, Oligochaeta and Turbellaria. The indication of stress refers to the NMDS performed on the lowest taxonomic level

The taxonomic composition of macroinvertebrate assemblages in leaf litter bags differed strongly between forested and non-forested sites (Fig. 2A; Table 2). The PEMANOVA supported strong evidence that the leaf-associated macroinvertebrate assemblages depended on the local riparian vegetation with EPT taxa more prevalent in the forested sites. There was no effect of either the region or the interaction between riparian vegetation and region (Table 2). Regional differences in macroinvertebrate assemblages were mostly related to the presence of Crustacea (mainly *Gammarus* sp.) in three of the four streams in the northern region, and their absence in all the southern streams (Fig. 2A). We found no statistically significant evidence for differences within EPT or shredder assemblages between either riparian vegetation or region (Table 2; Fig. 2B, C).

**Table 2:** PERMANOVA output for leaf-associated macroinvertebrate assemblages (DF = 1.13)

Assemblage	Variable	*F*	*R*^2^	*P* value
All				
	Riparian vegetation	1.62	0.09	**0.02**
	Region	3.35	0.19	0.14
	Riparian vegetation: region	0.51	0.03	0.83
EPT				
	Riparian vegetation	1.23	0.07	0.07
	Region	3.41	0.20	0.19
	Riparian vegetation: region	0.72	0.04	0.51
Shredder				
	Riparian vegetation	0.27	0.02	0.78
	Region	4.28	0.25	0.58
	Riparian vegetation: region	0.61	0.04	0.33
ACWMT				
	Riparian vegetation	1.27	0.09	0.27
	Region	1.23	0.08	0.72
	Riparian vegetation: region	0.08	0.01	0.98
BCWMT				
	Riparian vegetation	0.50	0.04	0.59
	Region	1.55	0.11	0.86
	Riparian vegetation: region	− 0.08	0.01	0.99

P-values < 0.05 are highlighted in bold

**Fig. 3:**
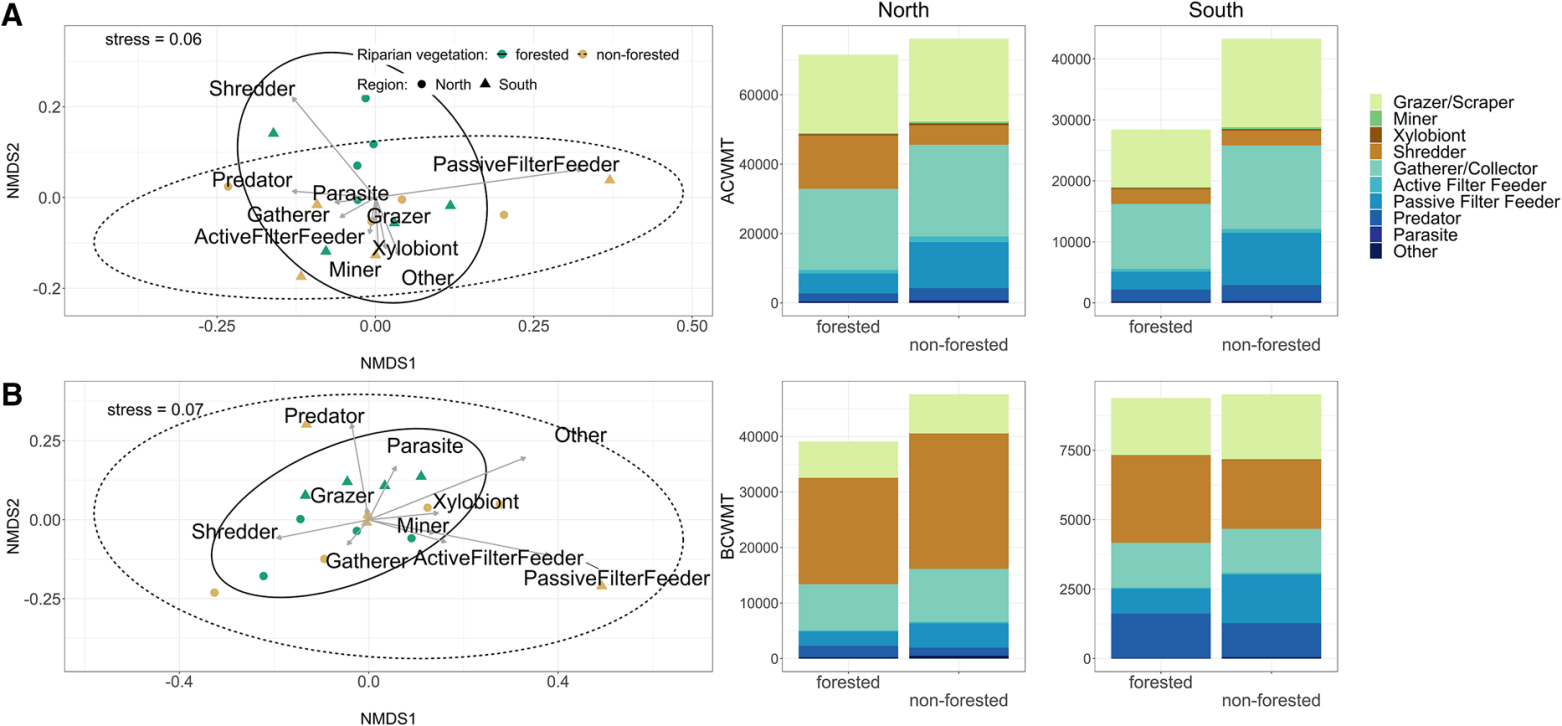
NMDS ordination (*n* = 16) and distribution of functional macroinvertebrate composition for **A** the community-weighted mean trait (CWMT) values based on abundance data (ACWMT) and **B** the CWMT values based on biomass (BCWMT). In the NMDS plots, green symbols indicate forested sites, brown symbols show non-forested sites, circles represent northern sites and triangles depict southern sites. Solid ellipses encircle communities from forested streams, and dashed ellipses enclose those from non-forested streams (95% confidence interval). The bar plots represent the cumulative number of values for each group

There was no evidence that the riparian vegetation or the region had an effect on either community-weighted composition (i.e., ACWMT and BCWMT) of the functional feeding groups (Table 2). However, high scores of ACWMT of the shredder group were associated with forested sites (Fig. 3A). The ACWMT proportions of the shredder group per leaf litter bag ranged from 0.63 to 42.77% (ACWMT mean proportions ± SD; north forested: 20.61 ± 8.96%; north non-forested: 8.60 ± 4.73%; south forested: 10.05 ± 9.95%; south non-forested: 5.57 ± 4.17%), which corresponded to absolute ACWMT values ranging from 5.11 to 3098.25 (ACWMT mean values ± SD; north forested: 481.66 ± 576.25; north non-forested: 175.24 ± 119.79; south forested: 78.47 ± 66.64; south non-forested 67.24 ± 86.07). BCWMT values of the shredder group represented a major part of the total macroinvertebrate biomass found in the leaf litter bags compared to other functional feeding groups (Fig. 3B). The mean BCWMT proportions of the shredder group per leaf litter bag ranged from 0.36 to 69.32% (BCWMT mean proportions ± SD; north forested: 43.66 ± 13.13%; north non-forested: 38.19 ± 22.00%; south forested: 29.06 ± 14.59%; south non-forested: 20.77 ± 15.96%), which corresponded to absolute BCWMT values ranging from 0.24 to 4768.83 (BCWMT mean values ± SD; north forested: 596.75 ± 589.90; north non-forested: 759.00 ± 1134.90; south forested: 101.31 ± 81.77; south non-forested 77.31 ± 92.63).

**Fig. 4:**
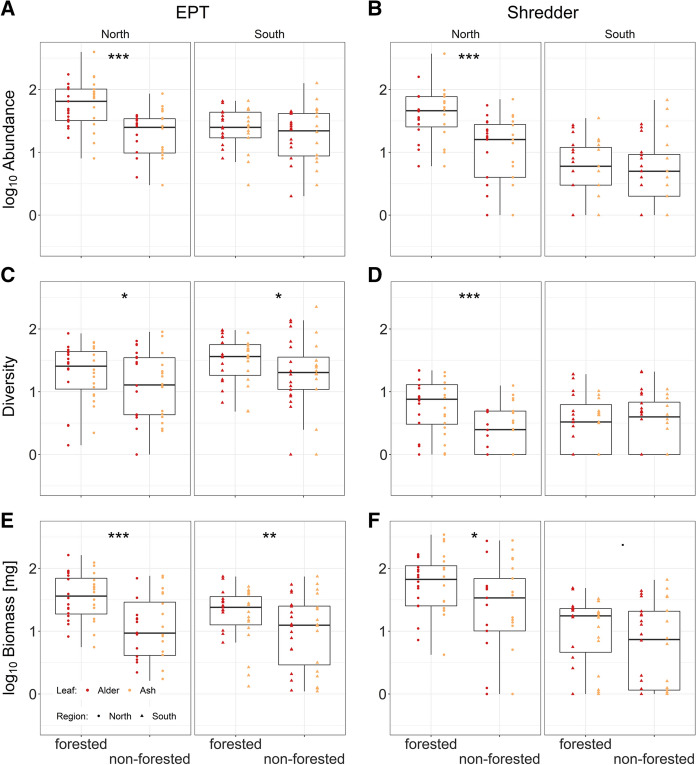
Boxplots of **A** EPT abundance, **B** shredder abundance, **C** EPT Shannon diversity, **D** shredder Shannon diversity, **E** EPT biomass and **F** shredder biomass per leaf litter bag between forested and non-forested riparian vegetation and between regions (red: alder; orange: ash; circles: north; triangles: south). Significance symbols: ‘***’ ≤ 0.001, ‘**’ ≤ 0.01, ‘*’ ≤ 0.05, ‘·’ ≤ 0.1

Abundance, Shannon diversity and biomass of EPT in the experimental leaf litter bags were higher in the forested sites compared to the non-forested sites in the northern region and Shannon diversity and biomass of EPT were also higher in the forested sites compared to the non-forested sites in the southern region (Fig. 4A, C, E; Table S2). We found strong evidence that EPT abundances were higher in the forested compared to the non-forested sites in the northern region (estimate = 0.52, *P* value < 0.01). However, our data also suggested that there were fewer EPT individuals in the leaf litter bags in the southern sites (estimate = −0.42, *P* value = 0.05), with an interaction effect between riparian vegetation and region (estimate = 0.41, *P* value < 0.01) due to a stronger effect of riparian vegetation in the northern compared to the southern region (Fig. 4A). The number of individuals belonging to EPT per leaf litter bag ranged from 1 to 676, meaning that all coarse leaf litter bags contained at least one individual belonging to EPT (mean abundance per bag ± SD: north forested: 97.06 ± 128.57; north non-forested: 23.88 ± 18.55; south forested: 25.55 ± 17.80; south non-forested: 25.66 ± 24.96).

Our data supported strong evidence for higher EPT Shannon diversity associated with the forested compared to non-forested sites (estimate = 0.19, *P* value = 0.05) with no effect of either region (estimate = 0.19, *P* value = 0.46) or interaction between riparian vegetation and region (estimate = 0.00, *P* value = 0.97; Fig. 4C). EPT Shannon diversity ranged from 0 to 2.35 (mean diversity per bag ± SD per bag: north forested: 1.29 ± 0.46; north non-forested: 1.10 ± 0.53; south forested: 1.49 ± 0.36; south non-forested: 1.29 ± 0.55), which corresponded to 1 to 13 EPT taxa per bag (mean richness per bag ± SD: north forested: 7.06 ± 1.87; north non-forested: 5.50 ± 2.71; south forested: 6.92 ± 1.92; south non-forested: 7.08 ± 3.36).

The data suggested strong evidence for higher EPT biomass in the forested compared to non-forested sites (estimate = 0.52, *P* value < 0.01). We found no statistically significant differences between the regions (estimate = −0.27, *P* value = 0.25) nor the interaction between riparian vegetation and the region (estimate = 0.24, *P* value = 0.11; Fig. 4E). EPT biomass per leaf litter bag ranged from 0.10 to 161.99 mg (mean biomass per bag ± SD per bag: north forested: 47.25 ± 38.15 mg; north non-forested: 18.89 ± 22.06 mg; south forested: 25.07 ± 18.02 mg; south non-forested: 18.03 ± 19.42 mg).

The abundance, Shannon diversity and biomass of shredders in the experimental leaf litter bags were higher in the forested sites compared to the non-forested sites in northern region, whereas in the southern region, only shredder biomass was slightly, yet statistically insignificantly higher in the forested sites compared to the non-forested sites (Fig. 4B, D, F; Table S2). Shredder abundance per leaf litter bag was higher in forested compared to non-forested sites in the northern region (estimate = 0.62, *P* value < 0.01). We also found lower shredder abundance in the south (estimate = − 0.86, *P* value = 0.01) and an interaction between riparian vegetation and region (estimate = 0.55, *P* value < 0.01) due to a more pronounced effect of riparian vegetation in the northern compared to the southern region (Fig. 4B). The abundance of shredders varied from 0 to 660 individuals per leaf litter bag (mean abundance per bag ± SD: north forested: 76.63 ± 125.17; north non-forested: 17.31 ± 16.86; south forested: 8.06 ± 8.40; south non-forested: 9.38 ± 14.95).

Shredder Shannon diversity was higher in forested compared to non-forested sites in the northern region (estimate = 0.37, *P* value < 0.01), and did not differ between the regions (estimate = −0.27, *P* value = 0.25). However, we found a strong effect of the interaction between riparian vegetation and region on the shredder Shannon diversity (estimate = 0.38, *P* value < 0.01) due to a stronger effect of riparian vegetation in the northern compared to the southern region (Fig. 4D). Shannon diversity of shredders ranged from 0 to 1.34 (mean diversity per bag ± SD: north forested: 0.76 ± 0.43; north non-forested: 0.39 ± 0.36; south forested: 0.50 ± 0.44; south non-forested: 0.51 ± 0.44), which corresponded to 0 to 6 shredder taxa per bag (mean richness per bag ± SD: north forested: 3.45 ± 1.12; north non-forested: 2.02 ± 1.10; south forested: 2.11 ± 1.14; south non-forested: 2.32 ± 1.12).

Shredders showed higher biomass in the forested sites compared to the non-forested sites in the northern region (estimate = 0.28, *P* value = 0.03), with marginally statistically insignificant differences between regions (estimate = −0.75, *P* value = 0.07) and no evidence for an interaction between riparian vegetation and region (estimate = 0.05, *P* value = 0.79). Shredder biomass ranged from 0 to 681.64 mg (mean biomass per bag ± SD: north forested: 87.87 ± 84.69 mg; north non-forested: 105.90 ± 163.30 mg; south forested: 15.64 ± 12.81 mg; south non-forested: 13.69 ± 17.35 mg) with a substantial contribution of Gammarid biomass (up to 678.13 mg) when these taxa occurred in the stream. There was strong evidence that abundance, diversity, and biomass correlated positively between the two groups of EPT and shredders (abundance: τ = 0.58, *P* value < 0.01; diversity: τ = 0.44, *P* value < 0.01; biomass: τ = 0.49, *P* value < 0.01).

### Leaf litter breakdown rates

Breakdown rates differed strongly between riparian vegetation types and leaf litter species (Table 3). Fragmentation rate *λ*_F_, was approximately three times higher in forested sites compared to non-forested sites within regions.

**Table 3:** Breakdown rates *k*_c_ (coarse), *k*_f_ (fine), *λ*_F_ (fragmentation), and mass loss values of the initial 5 g of leaf litter (means ± 1SD)

Region	Riparian vegetation	Leaf	*k*_c_ (mg dd^−1^)	Mass loss coarse (g)	*k*_f_ (mg dd^−1^)	Mass loss fine (g)	*λ*_F_ (mg dd^−1^)
North	Forested	Alder	5.69 ± 2.04	2.19 ± 0.59	3.41 ± 0.49	1.49 ± 0.18	1.27 ± 1.04
		Ash	11.13 ± 4.05	3.29 ± 0.66	6.17 ± 0.89	2.387 ± 0.29	2.76 ± 1.92
	Non-forested	Alder	4.15 ± 0.66	1.87 ± 0.17	3.35 ± 0.54	1.57 ± 0.16	0.42 ± 0.11
		Ash	7.43 ± 1.58	2.81 ± 0.23	5.56 ± 0.63	2.33 ± 0.22	0.99 ± 0.62
South	Forested	Alder	4.99 ± 0.90	2.11 ± 0.25	4.02 ± 0.68	1.78 ± 0.08	0.51 ± 0.30
		Ash	7.99 ± 1.91	2.89 ± 0.31	6.56 ± 0.74	2.57 ± 0.14	0.74 ± 0.56
	Non-forested	Alder	4.76 ± 0.79	2.03 ± 0.19	4.37 ± 0.66	1.90 ± 0.15	0.20 ± 0.05
		Ash	7.81 ± 0.87	2.88 ± 0.19	7.42 ± 0.75	2.79 ± 0.25	0.20 ± 0.21

There was strong evidence for differences in fragmentation rates between riparian vegetation types with substantially higher fragmentation rates in forested compared to non-forested sites irrespectively of whether the mixed effect model included shredder abundance, diversity, or biomass (estimate = 0.37/0.32/0.34, *P* value < 0.01/0.01/0.01, respectively; Fig. S2; Tables S4, S5). Ash showed higher fragmentation rates than alder (estimate = 0.21/0.22/0.22, *P* value = 0.01/0.02/0.01, respectively) and this leaf litter identity effect had similar effect sizes as riparian vegetation. There was no evidence that shredder abundance, diversity, or biomass had major effects on fragmentation rates (estimate = − 0.10/0.07/0.15, *P* value = 0.32/0.48/0.59, respectively) relative to the other factors such as type of the riparian vegetation or leaf treatment.

**Fig. 5:**
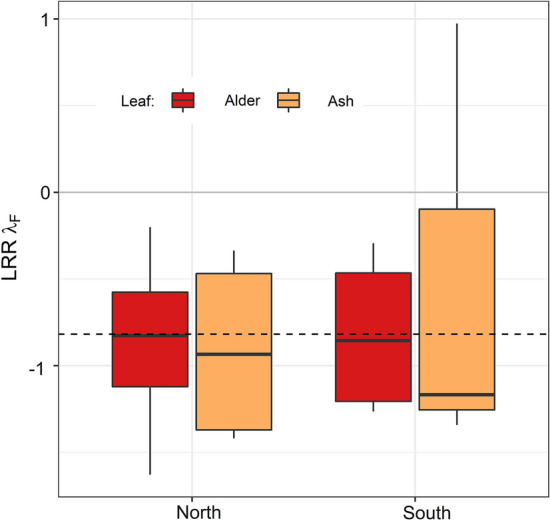
Mean log response ratio (LRR) of the fragmentation rate λ_F_ measured in the eight streams. Negative values indicate higher fragmentation rates in forested sites compared to non-forested sites of the same stream. LRR were calculated with mean values per site and leaf treatment (red: alder; orange: ash). The dashed line indicates the grand mean LRR of λ_F_

The average log response ratio (LRR) of *λ*_F_ measured in forested and non-forested sites showed negative values for seven of the eight studied streams indicating that the mean fragmentation rate was higher in forested sites compared to non-forested sites (Fig. 5). The random effect model showed that the mean LRR was clearly different from 0 and estimated at − 0.82 (SE = 0.35, CI [− 1.51, − 0.13], *P* value = 0.02).

## Discussion

Headwater streams harbor diverse aquatic communities, which are central for ecosystem processes, including leaf litter breakdown (Huryn et al., 2002; Clarke et al., 2008; Altermatt, 2013). In this study, we found distinct compositions of leaf-associated macroinvertebrate assemblages dependent on the vegetation type in the local riparian zone. Abundances of EPT and shredders were higher in forested compared to non-forested sites in streams in the northern, but not in the southern region. Diversity of EPT was higher in forested compared to non-forested sites in both regions, but shredder diversity only showed this pattern in the northern region. The biomass of EPT and shredders was higher in the forested compared to the non-forested sites though the statistical significance of the effect depended on region (higher in northern region). Furthermore, fragmentation rates were on average three times higher in forested compared to non-forested sites for both alder and ash. The effect size of riparian vegetation on fragmentation rates was comparable to the effect size of leaf treatment. Our findings demonstrate that the aquatic fauna and a key ecosystem process can strongly differ depending on the local vegetation type in the riparian zone.

Differences among macroinvertebrate assemblages emerged mainly due to higher prevalence of EPT and the shredder group in forested sites. A substantial part of these differences is based on the abundance of individuals from the order Plecoptera, which includes highly sensitive taxa with narrow ecological requirements (Küry, 1997; Zwick, 2000). One such requirement is the availability of CPOM, as many Plecoptera taxa belong to the functional feeding group of shredders. For example, taxa from the family Nemouridae have been shown to be efficient shredders in their larval stage (Jonsson & Malmqvist, 2000) and their microhabitat selection to be highly driven by CPOM availability and biomass (Ernst & Stewart, 1986; Piano et al., 2020). Leaves from the riparian vegetation not only fall in the streams and add to the CPOM stock (Tank et al., 2010), they also provide shelter for winged adults of many insect species on land (Harper, 1973; Yoshimura, 2012). However, in the leaf litter bags in the streams, the shredder group was not the most dominant feeding group in terms of community-weighted mean trait values based on abundance (ACWMT), which contrasts findings from other studies (Hieber & Gessner, 2002). Nevertheless, when CWMT was expressed based on biomass (BCWMT), the shredder group represented a dominant part of the functional macroinvertebrate assemblage composition in line with previous studies assessing the biomass of shredders in leaf litter bags (Tonin et al., 2014; Ferreira et al., 2016). These differences between ACWMT and BCWMT were caused by large-bodied shredders. In our experimental bags, taxa from the families of Limnephilidae or Gammaridae were not the most abundant but because of their large individuals, they contributed substantially to the total biomass of the communities. Our leaf litter bags were also colonized by many grazers/scrapers and gatherer/collectors, which typically feed on biofilm or deposited FPOM, respectively (Moog, 1995). These groups likely used the leaf litter as microhabitat and physical substrate rather than as a food source (Sanpera-Calbet et al., 2009). The input of allochthonous leaf litter from forests in the riparian zone may thus be beneficial to a variety of macroinvertebrates and can influence assemblages through additional ways besides food provision (O’Brien et al., 2017).

The overall effect of the presence of forests in the local riparian zone on EPT and shredder community metrics was positive, but the extent and significance of this effect varied between study regions. Expanding earlier studies (Stephenson & Morin, 2009; Iñiguez‐Armijos et al., 2016), our balanced and paired design of forested and non-forested stream sites highlights the favorable conditions provided by the presence of forests in the riparian zone on sensitive macroinvertebrates. Effects of the type of riparian vegetation on EPT and shredder community metrics were more pronounced in northern streams compared to southern streams, potentially due to the different geology and water chemistry (e.g., electrical conductivity; Table 1). The identity of macroinvertebrates and especially shredder taxa, with more responsive species in the northern streams, may have influenced the associations between community metrics and riparian vegetation type. As many individuals were too small to be determined on species level, species within the same genus from the northern region could have differed from species found in the southern Region. Another explanation for bigger differences in community metrics between forested and non-forested sites in the northern region could be in the river distance between forested and non-forested sites which were longer between the site pairs from the southern compared to the northern region (Table S1). Therefore, studies addressing gradients of riparian cover locally or on a catchment scale are useful to assess the spatial extent of the influence of the presence or absence of forests in the riparian zone (England & Rosemond, 2004; Death & Collier, 2010; Truchy et al., 2022). Lastly, leaf-associated macroinvertebrate assemblages could have been linked to microbial communities inhabiting and conditioning the leaves (Ferreira et al., 2016). It has been shown that macroinvertebrates can have feeding preferences for certain leaf-colonizing stream fungi that make the leaves more palatable (Arsuffi & Suberkropp, 1989), which could have compensated or enhanced differences associated with the riparian vegetation. Variation in the fungal communities, could be addressed by assessing their diversity and biomass (Bärlocher et al., 2010; Ferreira et al., 2016). Future studies on macroinvertebrate community patterns in headwater streams have much to gain from considering the local and regional conditions in the stream and its riparian vegetation and from simultaneously analyzing multiple community metrics from several organism groups.

In our study streams, fragmentation rates of leaf litter were mainly associated with leaf treatment and type of the riparian vegetation (forested vs. non-forested). Leaf characteristics such as nutrients and carbon content differ between leaves from different tree species, which influences the speed and process of leaf litter breakdown (Zhang et al., 2019). Therefore, it is not surprising that leaves of the two common riparian tree species alder and ash, that differ in these aspects (Hladyz et al., 2009; Bruder et al., 2014), also differed in their breakdown rates with ash leaves showing overall higher breakdown rates compared to alder. However, the breakdown rates also differed depending on the type of riparian vegetation with overall higher values in the forested compared to the non-forested section and with a comparable effect size as of leaf  treatment. However, we found no evidence for an effect of any of the shredder community metrics on fragmentation rates. This could be explained by the non-linear per capita feeding rate due to negative effects of competition (Little et al., 2019). Another explanation could lie in the time discrepancy between fragmentation rates which reflect the cumulative shredding activity over the entire duration of the experiment, and the shredder community present at the time at the end of the experiment. It is likely that we missed macroinvertebrate individuals, that had already fragmented and then left the leaf litter bags. Whether the remaining macroinvertebrates in the leaf litter bags were effectively shredding could only be examined by gut content or stable isotope analyses (Hall et al., 2000; Burdon et al., 2020a). Nevertheless, fragmentation rates were around three times higher in forested sites compared to non-forested sites for leaves from both tree species. Only the stream with the overall lowest fragmentation rates did not show this difference. The contribution of physical fragmentation and abrasion by water current was likely negligible as flow velocities in front of the leaf litter bags were substantially lower than reported threshold velocities for labile ash and alder (Table S1; Ferreira et al., 2006). The range of fragmentation rates (*λ*_F_) for both leaf treatments was comparable to the few studies that calculated fragmentation rates (Lecerf, 2017; Yeung et al., 2018; Omoniyi et al., 2021).

In conclusion, forests in the local riparian zone are key for the functioning of headwater streams. Biological integrity of river networks may depend on the vegetation type adjacent to the numerous streams that form their headwaters (Meyer et al., 2007). Our data suggest that the effects of the vegetation type in the local riparian zone can range from community to ecosystem level. We expect our results to contribute to and guide restoration strategies, specifically by highlighting the effects and value of natural riparian vegetation on indicator macroinvertebrate species and ecosystem processes. Maintaining and restoring forested headwater stream sections even on a local scale is crucial to conserve the characteristic fauna and important ecosystem processes in streams.

## Supplementary Information

Below is the link to the electronic supplementary material.

Supplementary file1 (DOCX 548 kb)

## Data Availability

The data is not publicly available as this study is part of an ongoing project for which final outputs were not yet released. However, the data is available from the authors upon request.
