# Supplementary material for: Leaf-associated macroinvertebrate assemblage and leaf litter breakdown in headwater streams depend on local riparian vegetation
Source: Hydrobiologia. 2022 Oct 18;850(15):3359–74. doi: 10.1007/s10750-022-05049-7 (PMC10307707; doi:10.1007/s10750-022-05049-7)
Supplement: Supplementary file 1 — Supplementary file1 (DOCX 548 kb) [file 10750_2022_5049_MOESM1_ESM.docx]

**Supplementary Material**

**Leaf-associated macroinvertebrate assemblage and leaf litter breakdown in headwater streams depend on local riparian vegetation**

Rebecca Oester _1,2,3_,*, Paula C. dos Reis Oliveira _4_, Marcelo S. Moretti _4_, Florian Altermatt _2,3_, Andreas Bruder _1_

**Associations**1 Institute of Microbiology, University of Applied Sciences and Arts of Southern Switzerland, via Flora Ruchat Roncati 15, CH-6850 Mendrisio, Switzerland

2 Department of Evolutionary Biology and Environmental Studies, University of Zurich, Winterthurerstr. 190, CH-8057 Zurich, Switzerland

3 Eawag: Swiss Federal Institute of Aquatic Science and Technology, Department of Aquatic Ecology, Überlandstrasse 133, CH-8600 Dübendorf, Switzerland

4 Laboratory of Aquatic Insect Ecology, Universidade Vila Velha, Av. Comissário José Dantas de Melo 21, 29102-920 Vila Velha, ES, Brazil

* Corresponding author: [rebecca.oester@supsi.ch](mailto:rebecca.oester@supsi.ch)


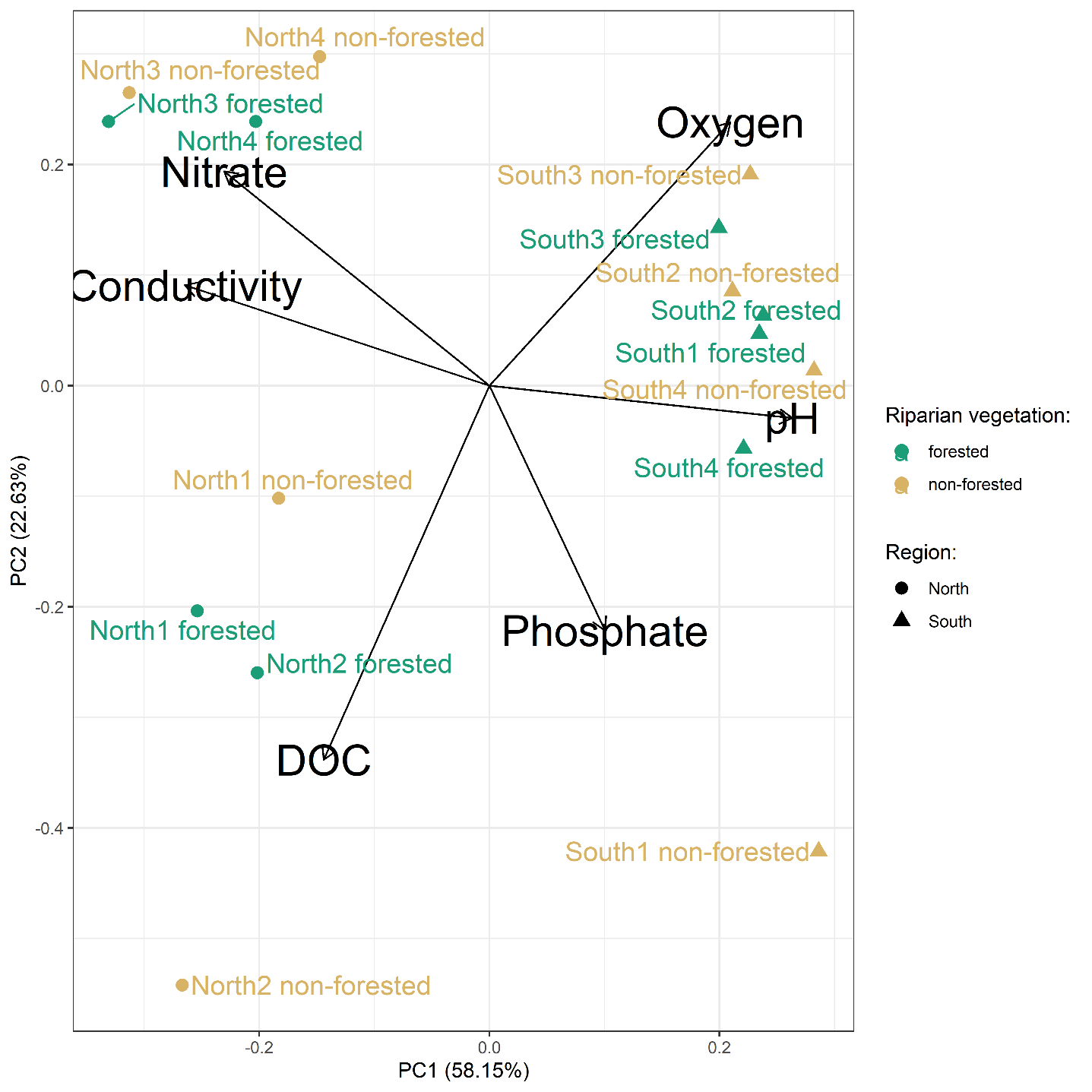


**Fig. S1** PCA for chemical variables measured at the 16 study sites


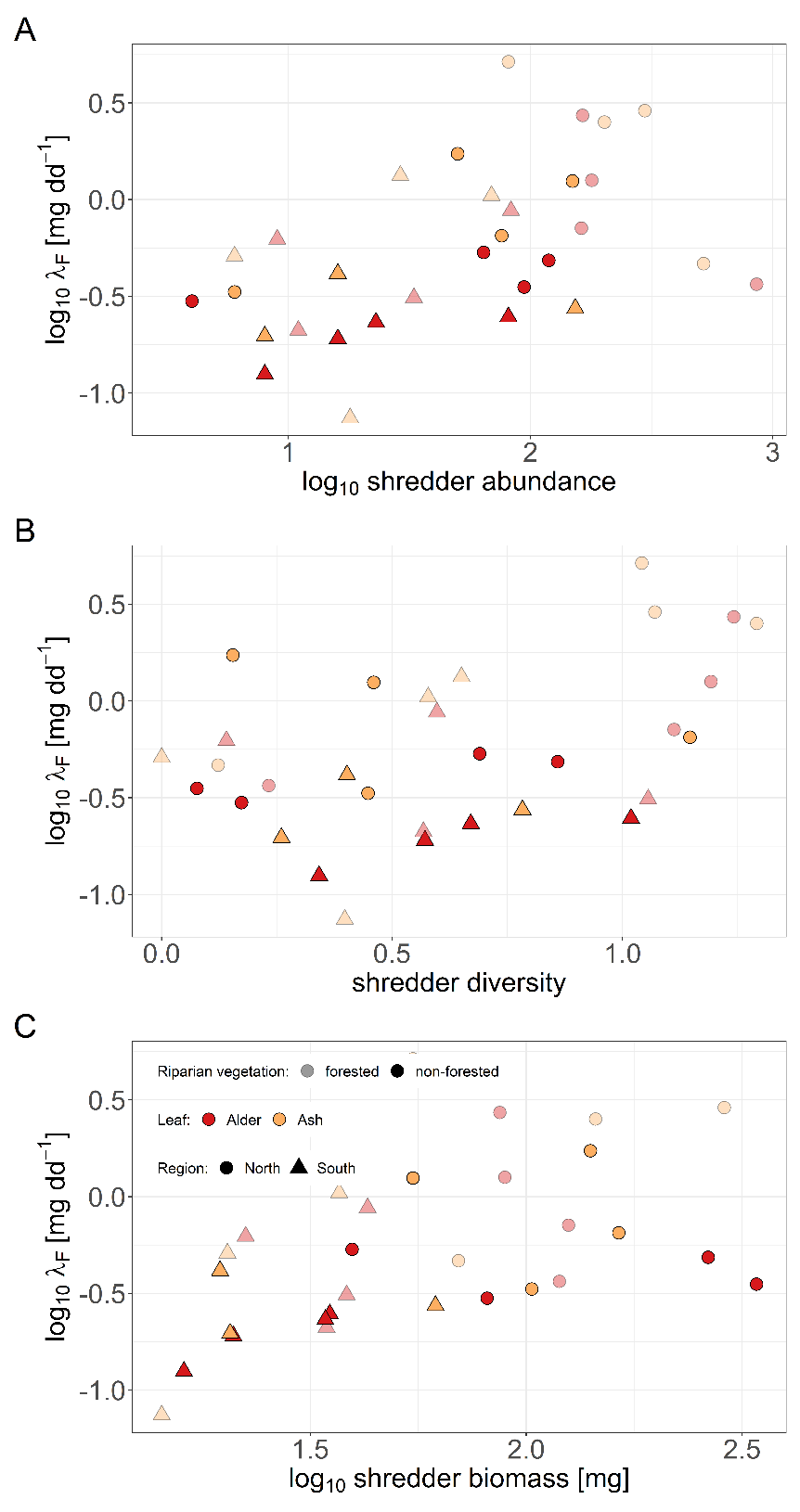


**Fig. S2** Fragmentation rate λ_F_ in relationship to shredder A) abundance, B) Shannon diversity and C) biomass. Symbols show mean values per site and leaf treatment (red: Alder; orange: Ash; circles: North; triangles: South; opaque: forested; solid: non-forested)

**Table S1** Site characterization of each study site

| Site | Riparian vegetation | Forest up-stream | Coordinates | Altitude [m a.s.l] | Catchment size [km^2^] | River distance [m] | Width [cm] | Depth [cm] | Velocity [cm/s] | Bag velocity [cm/s] | Average temperature [°C] | Nitrate [NO_3_^-^ mg/L] | Phosphate [PO_4_^3-^ µg/L] | DOC [mg/L] | pH | Oxygen [%] | Conductivity [µS/cm 20 °C] | Dominant substrates | Riparian cover [%] | Forest catchment [%] |
| --- | --- | --- | --- | --- | --- | --- | --- | --- | --- | --- | --- | --- | --- | --- | --- | --- | --- | --- | --- | --- |
| North1 | forested | YES | 47.53757, 9.27754 | 477.8 | 3.39 | 889 | 240.25 | 14.2 | 0.18 | 0.18 | 5.13 | 3.89 | 7.06 | 5.10 | 7.20 | 98.30 | 299 | Big Stones, Blocks, Sand | 29.56 | 14.61 |
|  | non-forested | YES | 47.54333, 9.27631 | 460.4 | 5.23 | 889 | 130.75 | 24.475 | 0.24 | 0.24 | 5.65 | 3.58 | 5.56 | 4.86 | 7.39 | 96.75 | 311 | Gravel, Small Stones, Big Stones | 15.06 | 19.52 |
| North2 | forested | NO | 47.57888, 9.35589 | 405.3 | 1.72 | 868 | 200 | 11.175 | 0.24 | 0.20 | 5.45 | 2.23 | 4.25 | 6.67 | 7.07 | 94.30 | 259 | Blocks,  Big Stones, Gravel | 29.12 | 83.12 |
|  | non-forested | NO | 47.57425, 9.35101 | 423.5 | 1.32 | 868 | 91.6 | 10.5 | 0.14 | 0.13 | 4.85 | 0.88 | 3.44 | 7.13 | 7.13 | 98.40 | 236 | Gravel, Small Stones, Blocks | 1.75 | 64.55 |
| North3 | forested | NO | 47.66457, 9.05680 | 481.6 | 0.97 | 378 | 199.05 | 9.375 | 0.13 | 0.09 | 5.80 | 6.14 | 2.38 | 1.87 | 7.00 | 97.95 | 390 | Gravel,  Big Stones, Small Stones | 29.33 | 38.17 |
|  | non-forested | NO | 47.66232, 9.05999 | 521.7 | 0.97 | 378 | 80.55 | 16.75 | 0.18 | 0.13 | 6.83 | 6.30 | 2.81 | 1.82 | 7.08 | 97.50 | 400 | Sand, Gravel, Litter | 11.56 | 38.17 |
| North4 | forested | YES | 47.64451, 8.84949 | 485.8 | 1.83 | 447 | 94.05 | 8.225 | 0.17 | 0.18 | 4.12 | 4.70 | 2.25 | 1.37 | 7.18 | 100.20 | 329 | Gravel, Small Stones, Litter | 36.38 | 27.81 |
|  | non-forested | YES | 47.64765, 8.84659 | 463.8 | 1.83 | 447 | 71.4 | 8.65 | 0.23 | 0.20 | 4.98 | 4.28 | 3.56 | 1.46 | 7.15 | 98.30 | 344 | Gravel, Small Stones,  Big Stones | 3.08 | 27.81 |
| South1 | forested | YES | 46.38287, 8.96934 | 410.8 | 1.60 | 380 | 179.3 | 11.5 | 0.19 | 0.18 | 4.09 | 0.49 | 2.56 | 1.73 | 8.15 | 98.70 | 62 | Blocks, Small Stones,  Big Stones | 30.39 | 95.04 |
|  | non-forested | YES | 46.37961, 8.96955 | 349.6 | 1.60 | 380 | 107.85 | 15.2 | 0.46 | 0.47 | 3.78 | 0.49 | 27.88 | 2.17 | 8.15 | 100.20 | 66 | Blocks,  Big Stones, Gravel | 0.25 | 95.04 |
| South2 | forested | YES | 46.44448, 8.93261 | 760.9 | 1.37 | 365 | 265.6 | 19.1 | 0.15 | 0.07 | 3.15 | 0.63 | 5.19 | 1.11 | 8.06 | 101.90 | 68 | Small Stones,  Big Stones, Blocks | 35.60 | 72.83 |
|  | non-forested | YES | 46.44661, 8.93478 | 700.0 | 3.32 | 365 | 351.85 | 17.025 | 0.28 | 0.10 | 3.09 | 0.78 | 11.63 | 1.07 | 7.55 | 100.25 | 108 | Blocks, Big Stones, Gravel | 5.32 | 59.89 |
| South3 | forested | NO | 46.23655, 9.02006 | 248.5 | 6.07 | 262 | 559.05 | 13.45 | 0.26 | 0.16 | 3.28 | 0.80 | 1.50 | 1.14 | 7.93 | 101.65 | 82 | Big Stones, Blocks, Gravel | 27.71 | 83.40 |
|  | non-forested | NO | 46.23440, 9.02092 | 262.9 | 6.07 | 262 | 402.35 | 18.3 | 0.26 | 0.09 | 3.34 | 0.80 | 2.13 | 1.11 | 7.88 | 100.55 | 79 | Blocks, Sand, Big Stones | 2.80 | 83.40 |
| South4 | forested | NO | 46.27369, 9.00879 | 248.7 | 3.57 | 265 | 215.35 | 10.75 | 0.23 | 0.14 | 2.67 | 0.72 | 5.44 | 2.23 | 8.09 | 101.30 | 31 | Gravel, Sand,  Litter | 22.68 | 49.64 |
|  | non-forested | NO | 46.27457, 9.00759 | 249.2 | 3.57 | 265 | 235.7 | 19.4 | 0.09 | 0.10 | 2.83 | 0.73 | 5.94 | 2.29 | 8.19 | 99.60 | 30 | Gravel, Litter, Small Stones | 13.78 | 49.64 |

**Table S2** Summary output from linear mixed-effect models of EPT and shredder abundance, diversity, and biomass. The intercept corresponds to expected log_10_(abundance+1)/diversity/log_10_(biomass+1) per bag located in a forested site in a North stream. Estimates represent changes in the slope from forested to non-forested and from North to South respectively. Marginal R^2^ represents the variance explained by the fixed factors, conditional R^2^ represents the variance explained by the entire model including both fixed and random effects. Statistically significant values are highlighted in bold

|  | Fixed Effects | | | | | Random Effects | | |  |
| --- | --- | --- | --- | --- | --- | --- | --- | --- | --- |
|  | Intercept | Riparian Vegetation [non-forested] | Region [South] | Riparian Vegetation [non-forested]: Region[South] | Marginal R^2^ | | SD Intercept Stream | SD Residual Stream | Conditional R^2^ |
| EPT Abundance | DF=117 |  |  |  |  | |  |  |  |
| Est | 1.79 | -0.52 | -0.42 | 0.41 | 0.23 | | 0.20 | 0.34 | 0.44 |
| SE | 0.12 | 0.08 | 0.17 | 0.12 |  | |  |  |  |
| t | 15.09 | -6.19 | -2.50 | 3.39 |  | |  |  |  |
| p | **<0.01** | **<0.01** | **0.05** | **<0.01** |  | |  |  |  |
| EPT  Diversity | DF=117 |  |  |  |  | |  |  |  |
| Est | 1.29 | -0.19 | 0.20 | <-0.01 | 0.06 | | 0.33 | 0.37 | 0.48 |
| SE | 0.18 | 0.09 | 0.26 | 0.13 |  | |  |  |  |
| t | 7.07 | -1.99 | 0.77 | -0.04 |  | |  |  |  |
| p | **<0.01** | **0.05** | 0.47 | 0.97 |  | |  |  |  |
| EPT Biomass | DF=117 |  |  |  |  | |  |  |  |
| Est | 1.54 | -0.52 | -0.26 | 0.24 | 0.17 | | 0.26 | 0.42 | 0.39 |
| SE | 0.15 | 0.11 | 0.21 | 0.15 |  | |  |  |  |
| t | 10.44 | -4.92 | -1.29 | 1.59 |  | |  |  |  |
| p | **<0.01** | **<0.01** | 0.24 | 0.11 |  | |  |  |  |
| Shredder Abundance | DF=117 |  |  |  |  | |  |  |  |
| Est | 1.63 | -0.62 | -0.86 | 0.55 | 0.34 | | 0.32 | 0.40 | 0.60 |
| SE | 0.17 | 0.10 | 0.25 | 0.14 |  | |  |  |  |
| t | 9.35 | -6.17 | -3.49 | 3.85 |  | |  |  |  |
| p | **<0.01** | **<0.01** | **0.01** | **<0.01** |  | |  |  |  |
| Shredder  Diversity | DF=117 |  |  |  |  | |  |  |  |
| Est | 0.76 | -0.37 | -0.67 | 0.38 | 0.09 | | 0.28 | 0.34 | 0.45 |
| SE | 0.15 | 0.08 | 0.21 | 0.12 |  | |  |  |  |
| t | 5.04 | -4.36 | -1.25 | 3.18 |  | |  |  |  |
| p | **<0.01** | **<0.01** | 0.26 | **<0.01** |  | |  |  |  |
| Shredder Biomass | DF=117 |  |  |  |  | |  |  |  |
| Est | 1.75 | -0.28 | -0.75 | 0.05 | 0.24 | | 0.43 | 0.52 | 0.55 |
| SE | 0.24 | 0.13 | 0.33 | 0.19 |  | |  |  |  |
| t | 7.50 | -2.15 | -2.28 | 0.27 |  | |  |  |  |
| p | **<0.01** | **0.03** | 0.06 | 0.78 |  | |  |  |  |

**Table S3** Anova (type III SS) outputs for EPT and shredder community metrics from linear mixed-effect models

| Variables | Chisq | p-value |
| --- | --- | --- |
| EPT abundance |  |  |
| Intercept | 227.63 | **<0.01** |
| Riparian Vegetation | 38.27 | **<0.01** |
| Region | 6.25 | **0.01** |
| Riparian Vegetation:Region | 11.48 | **<0.01** |
|  |  |  |
| EPT diversity |  |  |
| Intercept | 49.95 | **<0.01** |
| Riparian Vegetation | 3.97 | **0.05** |
| Region | 0.60 | 0.44 |
| Riparian Vegetation:Region | <0.01 | 0.97 |
|  |  |  |
| EPT biomass |  |  |
| Intercept | 108.89 | **<0.01** |
| Riparian Vegetation | 24.22 | **<0.01** |
| Region | 1.65 | 0.20 |
| Riparian Vegetation:Region | 2.53 | 0.11 |
| Shredder abundance |  |  |
| Intercept | 87.42 | **<0.01** |
| Riparian Vegetation | 38.10 | **<0.01** |
| Region | 12.19 | **<0.01** |
| Riparian Vegetation:Region | 14.86 | **<0.01** |
|  |  |  |
| Shredder diversity |  |  |
| Intercept | 25.38 | **<0.01** |
| Riparian Vegetation | 19.06 | **<0.01** |
| Region | 1.57 | 0.21 |
| Riparian Vegetation:Region | 10.13 | **<0.01** |
|  |  |  |
| Shredder biomass |  |  |
| Intercept | 56.32 | **<0.01** |
| Riparian Vegetation | 4.63 | **0.03** |
| Region | 5.20 | **0.02** |
| Riparian Vegetation:Region | 0.07 | 0.78 |

**Table S4** Summary output of the linear mixed-effect models for fragmentation rate with shredder abundance, diversity, and biomass. The intercept corresponds to expected log_10_ fragmentation rates an alder bag located in a non-forested site in the North region. Estimates represent changes in the slope from forested to non-forested, from North to South and from alder to ash respectively. Marginal R^2^ represents the variance explained by the fixed factors, conditional R^2^ represents the variance explained by the entire model including both fixed and random effects. Statistically significant values are highlighted in bold

|  | Fixed Effects | | | | | Random Effects | | |  | |  | |
| --- | --- | --- | --- | --- | --- | --- | --- | --- | --- | --- | --- | --- |
|  | Intercept | Shredder Community metric | Riparian Vegetation [non-forested] | Region [South] | Leaf [Ash]] | | Marginal R^2^ | SD Intercept Stream | SD Residual Stream | Conditional R^2^ | |  |
| Fragmentation rate | DF=20 | Abundance |  |  |  | |  |  |  |  | |  |
| Est | -2.76 | -0.10 | -0.38 | -0.51 | 0.21 | | 0.43 | 0.27 | 0.22 | 0.78 | |  |
| SE | 0.27 | 0.10 | 0.09 | 0.22 | 0.79 | |  |  |  |  | |  |
| t | -10.04 | -1.01 | -4.27 | -2.31 | 2.66 | |  |  |  |  | |  |
| p | **<0.01** | 0.32 | **<0.01** | 0.06 | **0.01** | |  |  |  |  | |  |
| Fragmentation rate | DF=20 | Diversity |  |  |  | |  |  |  |  | |  |
| Est | -3.06 | 0.08 | -0.32 | -0.41 | 0.22 | | 0.47 | 0.23 | 0.23 | 0.73 | |  |
| SE | 0.17 | 0.11 | 0.09 | 0.18 | 0.08 | |  |  |  |  | |  |
| t | -17.72 | 0.73 | -3.65 | -2.22 | 2.63 | |  |  |  |  | |  |
| p | **<0.01** | 0.48 | **<0.01** | 0.07 | **0.02** | |  |  |  |  | |  |
| Fragmentation rate | DF=20 | Biomass |  |  |  | |  |  |  |  | |  |
| Est | -3.30 | 0.15 | -0.34 | -0.35 | 0.22 | | 0.45 | 0.25 | 0.22 | 0.75 | |  |
| SE | 0.60 | 0.28 | 0.08 | 0.26 | 0.08 | |  |  |  |  | |  |
| t | -5.54 | 0.55 | -4.13 | -1.35 | 2.66 | |  |  |  |  | |  |
| p | **<0.01** | 0.59 | **<0.01** | 0.23 | **0.01** | |  |  |  |  | |  |

**Table S5** Anova (type III SS) outputs for the fragmentation rates from linear mixed-effect models

| Variables | Chisq | p-value |
| --- | --- | --- |
| Intercept | 100.71 | **<0.01** |
| Shredder Abundance | 1.02 | 0.31 |
| Riparian Vegetation | 18.24 | **<0.01** |
| Region | 5.31 | **0.02** |
| Leaf | 7.10 | **<0.01** |
| Intercept | 313.82 | **<0.01** |
| Shredder Diversity | 0.53 | 0.47 |
| Riparian Vegetation | 13.33 | **<0.01** |
| Region | 4.94 | **0.03** |
| Leaf | 6.92 | **<0.01** |
| Intercept | 30.65 | **<0.01** |
| Shredder Biomass | 0.31 | 0.58 |
| Riparian Vegetation | 17.04 | **<0.01** |
| Region | 1.83 | 0.18 |
| Leaf | 7.09 | **<0.01** |
